# Using large language models to learn from recent climate change discourse in public health

**DOI:** 10.1371/journal.pone.0321309

**Published:** 2025-04-29

**Authors:** Anna Belova, Raquel A. Silva, Dylan M. Vorndran, Natalie R. Sampson

**Affiliations:** 1 ICF Inc, Fairfax, Virginia, United States of America; 2 University of Michigan-Dearborn, Dearborn, Michigan, United States of America; University of California Davis, UNITED STATES OF AMERICA

## Abstract

**Background:**

Public health has increasingly recognized the links between climate change and health, emphasizing the need to address related inequities. This is reflected in work led by the Intergovernmental Panel on Climate Change, the UN Framework Convention on Climate Change, the U.S. National Climate Assessment, and leading health-related professional associations, such as the American Public Health Association (APHA). We ask how the focus of climate change-related topics in public health discourse has evolved, and what does this signal about the field’s role and capacity to address this global crisis?

**Methods:**

We analyzed close to 41,000 abstracts from APHA annual meetings (2017–2023). Using a combination of large language models and expert review, we identified and analyzed over 1,100 abstracts with climate change-related content. We used a fine-tuned OpenAI GPT-3.5 model to detect abstracts with climate change-related content and the Claude 3.0 Sonnet model to categorize these abstracts into 21 themes and 12 health outcome categories.

**Results:**

Since 2017, the discussion of climate change at APHA has declined both in terms of volume and topic diversity. The impacts of climate change on heat-related illness, stress and mental illness, and vector-borne diseases were the most common topics discussed. Fewer abstracts discussed the role of public health, workforce development, and policy and advocacy, with slightly more attention focused on health communication and education.

**Conclusions:**

Although this is only a snapshot of recent discourse in the field, trends suggest the need to build capacity for climate action. Addressing the climate crisis is not solely an environmental health issue; it is a public health issue. Advocates, policymakers, and scholars know that innovative and intersectoral solutions are critical for effective and equitable climate action. However*, within* public health, we must work together and jointly contribute to reducing the unequal and extensive burdens associated with our changing climate.

## STARTLEMENT

It is a forgotten pleasure, the pleasure

of the unexpected blue-bellied lizard

skittering off his sun spot rock, the flicker

of an unknown bird by the bus stop.

To think, perhaps, we are not distinguishable

and therefore no loneliness can exist here.

Species to species in the same blue air, smoke—

wing flutter buzzing, a car horn coming.

So many unknown languages, to think we have

only honored this strange human tongue.

If you sit by the riverside, you see a culmination

of all things upstream. We know now,

we were never at the circle’s center, instead

all around us something is living or trying to live.

The world says, What we are becoming, we are

becoming together.

The world says, One type of dream has ended

and another has just begun.

The world says, Once we were separate,

and now we must move in unison.

- Ada Limón, 24th Poet Laureate Consultant in Poetry at the Library of Congress, written for the Fifth National Climate Assessment.

## Introduction

Climate change is among society’s most urgent issues. It is public health’s “catastrophe in waiting.” [1] Globally, we are already experiencing the health-related effects of climate change. These include, for instance, the impacts of extreme weather events, such as heat-related illnesses and injury, shifts in the distribution of vector-borne diseases, more intense pollen production with impacts on respiratory health, and population displacement for coastal and island communities, among many, many others. Current and anticipated impacts of climate change on health are especially pronounced in communities burdened by historically discriminatory policies. In our changing climate, environmental racism has led to lower environmental quality, lack of community investment, and greater social vulnerability. [2,3] Increasingly, over the last few decades, public health has recognized the links between climate change and health and the need to address related inequities. [4]In its 2007 Fourth Assessment Report, the Intergovernmental Panel on Climate Change (IPCC) began prominently addressing the health impacts of climate change. [5] This report marked a significant step in the IPCC’s recognition and documentation of the various ways in which climate change affects human health, raising global awareness of these connections. Subsequent IPCC reports have continued to emphasize and expand on the review of these health impacts, highlighting the urgent need for global action to mitigate climate change and adapt to its consequences in order to protect public health. [6,7]

Also at the global level, the United Nations (UN), through the UN Framework Convention on Climate Change, continues to organize international climate negotiations at annual Conference of the Parties (COP) meetings. Perhaps most well-known, COP 21, held in Paris in 2015, resulted in the adoption of the Paris Agreement, a landmark international treaty aimed at addressing climate change. The Paris Agreement set ambitious goals to limit global warming well below 2 degrees Celsius above pre-industrial levels and included commitments from nearly all countries to reduce greenhouse gas emissions. More recently, in November 2021, COP 26 marked a belated but significant milestone with its explicit emphasis on justice and the development of climate-resilient healthcare systems. For the first time, a dedicated pavilion focused solely on climate and health was established, signaling a shift towards integrating health considerations into broader climate policy frameworks.

In the U.S., the National Climate Assessments (NCA) began documenting climate change and projections in 2000, but it was not until 2014 that significant attention was given to the health impacts of climate change. This report included a dedicated chapter on “Human Health,” highlighting how climate change affects human health through various pathways, such as heat-related illnesses, air quality impacts, vector-borne diseases, and mental health effects. [8] The most recent Fifth National Climate Assessment, published in 2023, has full chapters on several exposures (e.g., air quality) and human health, and regions of the U.S. with localized public health threats associated with climate projections described for respective regions. [9]Mirroring this increased attention to climate as a public health issue, the American Public Health Association (APHA)— the leading professional organization in the public health field in the U.S. with over 25,000 members—held its 2017 annual meeting with the theme ‘Climate Changes Health,’ [10] launched a Center for Climate, Health and Equity in 2019, [11] and held its first-ever Climate, Health and Equity Summit in 2024. [12] This virtual summit began with a keynote by Dr. John Balbus, Director of the Office of Climate Change and Health Equity in the U.S. Department of Health and Human Services, who emphasized the interconnectedness of climate change and social determinants of health, stressing the urgency for cross-sector and multi-solving approaches. This event was thirty years after the first-ever panel session on climate change at an APHA annual meeting in 1994.

Advocates, policymakers, and scholars know that innovative and intersectoral solutions are critical to effective and equitable climate action. Within the field of public health, we know we must all contribute to reducing the unequal and extensive burdens associated with our changing climate. In this study, we aim to characterize and reflect on the recent evolution of public health discourse surrounding climate change impacts, challenges, and solutions. We do this by analyzing the discourse at APHA’s annual meetings, as these meetings are attended by thousands of public health researchers, practitioners, and advocates each year. To address the challenge of reviewing tens of thousands of abstracts accepted for presentation in recent years, we leveraged large language models to assist our analysis.

## Materials and Methods

We analyzed narratives of 40,967 abstracts accepted at the APHA annual meetings from 2017 to 2023 using a combination of manual review and large language models. The abstract collection was retrieved in August 2023; it does not include the late-breaking abstracts in the APHA 2023 or abstracts in the APHA 2024 program. APHA has 33 sections, with each section setting its own program. Sections are allotted a specific number of sessions based on the size of their membership, ranging from a few to 40 or more. Additionally, APHA staff, caucuses, and unique circumstances may lead to several additional sessions on the annual program.

We identified “climate change”-related abstracts based on the abstract text, abstract title, and session title using an OpenAI GPT 3.5-turbo-1106 model fine-tuned on manually tagged APHA 2017 and APHA 2022 abstracts. [13] Climate change was the theme of the APHA 2017 meeting, marking an awareness of the relationship between climate and health in the public health community. The APHA 2022 meeting marked the 5th anniversary of this initial focus. We reviewed the model predictions for APHA 2018–2021 and 2023 and determined the model precision to be 90.3%. Overall, we identified 1,157 climate-related abstracts among the abstracts accepted at APHA 2017–2023. Of those, 22% only mentioned climate change in passing, while 15% focused on related topics (e.g., health impacts of using fossil fuels or renewable energy sources, health effects of wildfires, sustainable food systems, and dietary patterns) but did not make an explicit connection between the topic and climate change. Next, taking the set of 1,157 abstracts determined to be climate-related, we used the Claude 3.0 Sonnet model developed by Anthropic to perform a variety of tasks. [14]

First, we applied the model to determine the common themes across the entire set of abstracts Using our subject matter expertise, we manually refined the initial output into a list of 21 themes. We then had the model assign applicable themes to each of the 1,157 abstracts. In many cases, the model assigned multiple themes to any given abstract. We reviewed the output to detect abstracts that were not assigned at least one theme and reprocessed the abstracts missing an assigned theme through the model. This iterative process continued until every abstract was assigned one or more themes. Finally, we conducted spot checks on a sample of abstracts to ensure the accurate assignment of themes by the model.

Second, using a similar approach, we applied the model to determine the health outcomes discussed in the climate-related abstracts. Using our subject matter expertise, we curated the initial output into a list of 12 common health outcomes found across the set of abstracts. After adding a “No health outcomes” category to account for abstracts that did not discuss health outcomes, we had the model assign applicable health outcomes categories to each of the 1,157 abstracts. Similar to the theme analysis, in some cases, the model assigned multiple health outcomes to any given abstract. We reviewed the output to identify abstracts that were not assigned to either a health outcome category or the “‘No health outcomes” category. These abstracts were reprocessed through the model. This process was repeated until every abstract was either assigned one or more health outcome categories or explicitly determined to have no discussion of health outcomes. Finally, as in the theme analysis, we conducted spot checks on a sample of abstracts to verify the accurate assignment of health outcomes by the model.

Lastly, we evaluated annual trends in program diversity for the climate-related abstracts. Program diversity refers to the extent to which multiple programs contribute to the body of climate-related abstracts, with greater diversity indicating a broader representation of programs. To measure diversity, we used the Herfindahl–Hirschman Index (HHI), [15] which quantifies the concentration of abstract contributions across programs. The HHI is calculated as ∑_*i*=1_^*N*^(*s*_*i*_)^2^, where *s*_*i*_ is the share of abstracts from program *i* and *N* is the total number of contributing programs. The HHI ranges from 0.01 (maximum diversity, the same number of abstracts is contributed by each of the 74 APHA programs) to 1 (no diversity, all abstracts are contributed by a single program). The HHI is commonly used in economics to measure market concentration.

## Results

**Fig 1:**
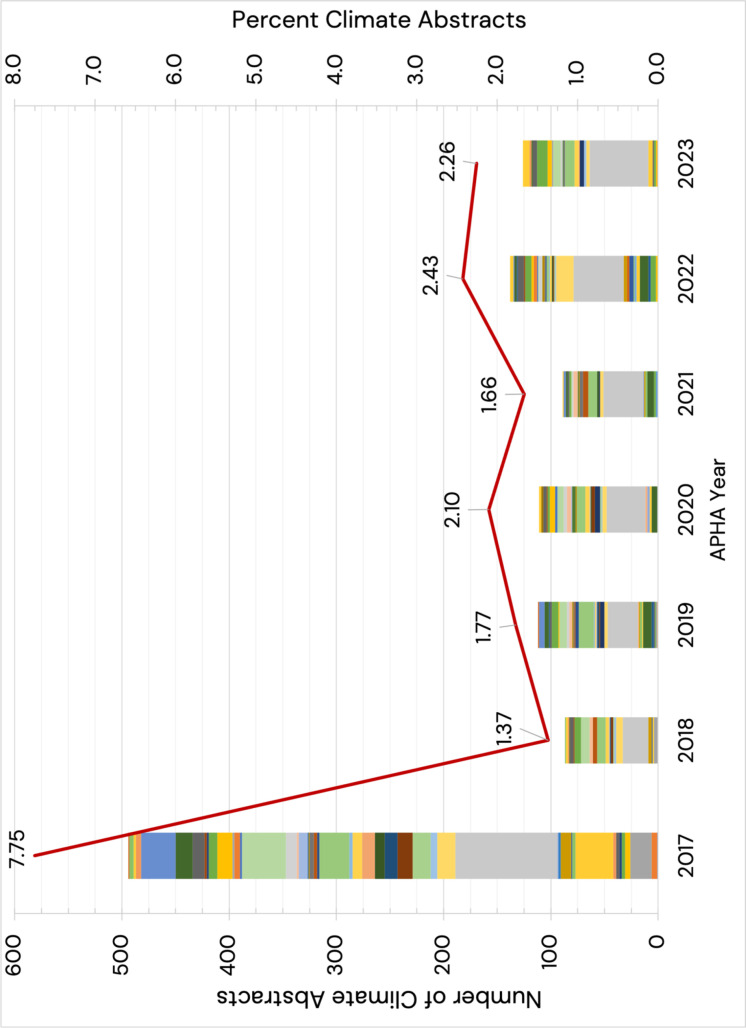
Climate change-related abstracts, APHA 2017-2023. Percentage, number, and program diversity (measured using the HHI) of climate change-related abstracts.

Fig 1 shows the time trends in the number of accepted APHA abstracts on climate change topics. APHA 2017 included 494 (7.7% of total) abstracts on climate change topics; this was the largest number of abstracts in the 2017–2023 period. In 2018, the number of climate change-related abstracts dropped to 87 (1.4% of total), rising to close to 126 abstracts (2.6% of total) in 2023. The diversity of programs contributing abstracts on climate-related topics also decreased over time. As measured by the HHI, the program diversity was greatest in 2017 (HHI of 0.07): 25 of 74 programs included at least five abstracts, and 16 programs included 10 or more abstracts. During 2018–2023, the program diversity declined (HHI of 0.15 on average across years).

The Environment Section program was the top program in terms of the number of abstracts on climate change topics: 322 out of 1,061 abstracts from the program during 2017–2023. This Section formed a climate change and health topic committee in the early 2000s and spearheaded the creation of APHA’s Intersectional Council topical group on Climate Change and Health in 2019. As expected, since its inception, APHA’s Center for Climate, Health and Equity program focused on climate and health topics, but the total number of abstracts was much smaller: 27 out of 35 abstracts from the program during 2017–2022. Other programs showed a varied number and proportion of climate change-related abstracts over time. Again, our abstract collection does not capture 2023 abstracts from APHA’s Center for Climate, Health and Equity. These late-breaking abstracts were added to the program after August 2023 (i.e., after our data were retrieved).

**Fig 2:**
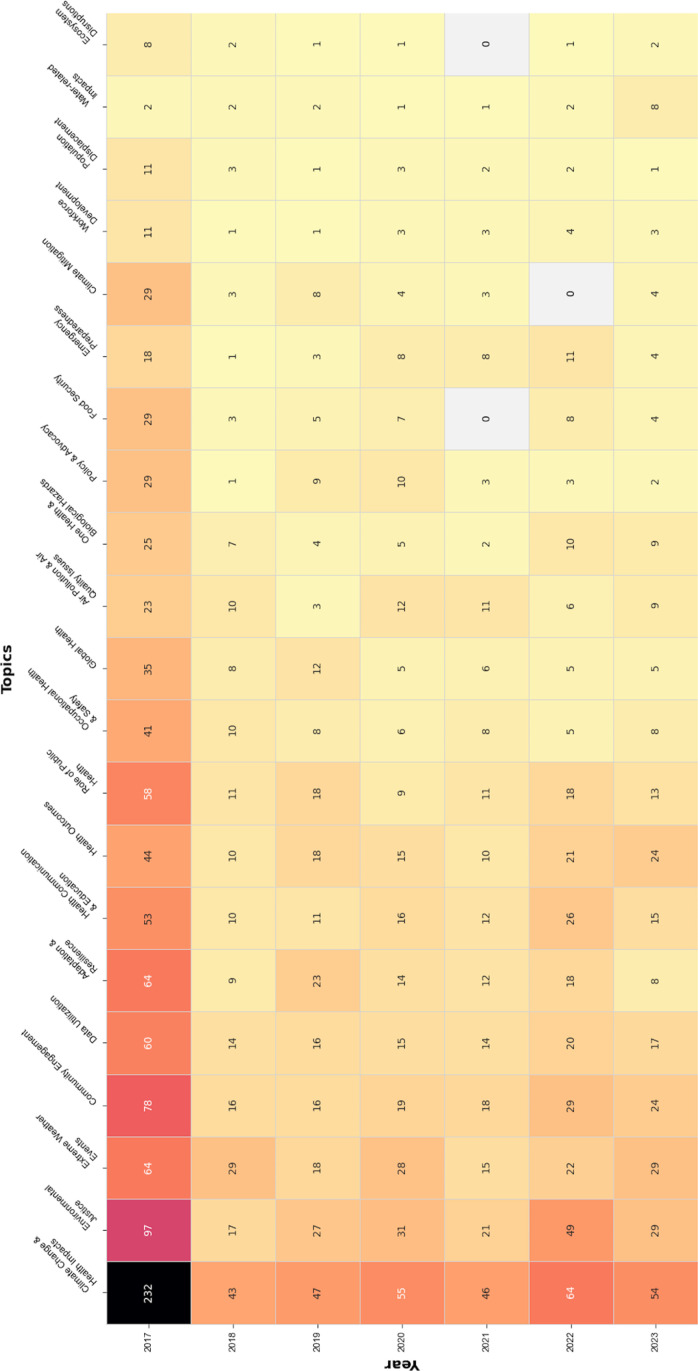
Heat map, APHA 2017-2023 climate change related abstracts by topic.

Examining the content of climate change-related abstracts during the 2017–2023 period (see Fig 2), 21 themes emerged. Both in 2017 and over time, *climate change & health impacts* dominated the discourse, followed by *environmental justice* and *community engagement*. These themes were present in most programs. Other themes with a large presence were *extreme weather events* and *adaptation & resilience*, as well as *data utilization*, *health communication & education*, and *the role of public health*.

**Fig 3:**
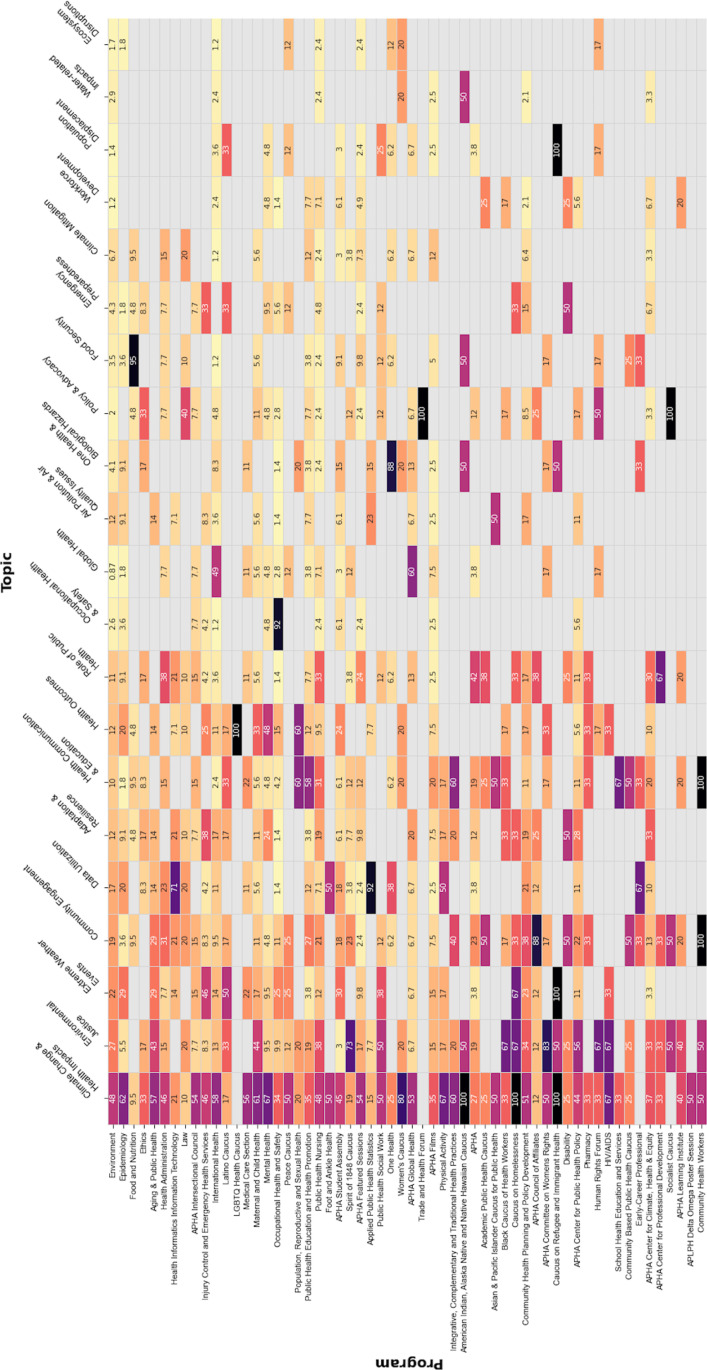
Heat map, APHA 2017-2023 climate change-related abstracts by program.

Considering the programs that included at least one climate change-related abstract, not all themes were broadly discussed across programs (see Fig 3). While the top themes (*climate change & health impacts, environmental justice,* and *community engagement*) were present in most programs, the remaining themes were more aligned with the focus area of each program. For example, *data utilization* was covered in 92% and 71% of the climate-related abstracts of the Applied Public Health Statistics and the Health Informatics Information Technology programs, respectively, and not covered by many programs; *occupational health & safety* was covered by 92% of the climate-related abstracts of the Occupational Health and Safety program but not covered at all by most other programs; *food security* dominated the Food and Nutrition program; *population displacement* was discussed by all climate-related abstracts included in the Caucus on Refugee and Immigrant Health program.

**Fig 4:**
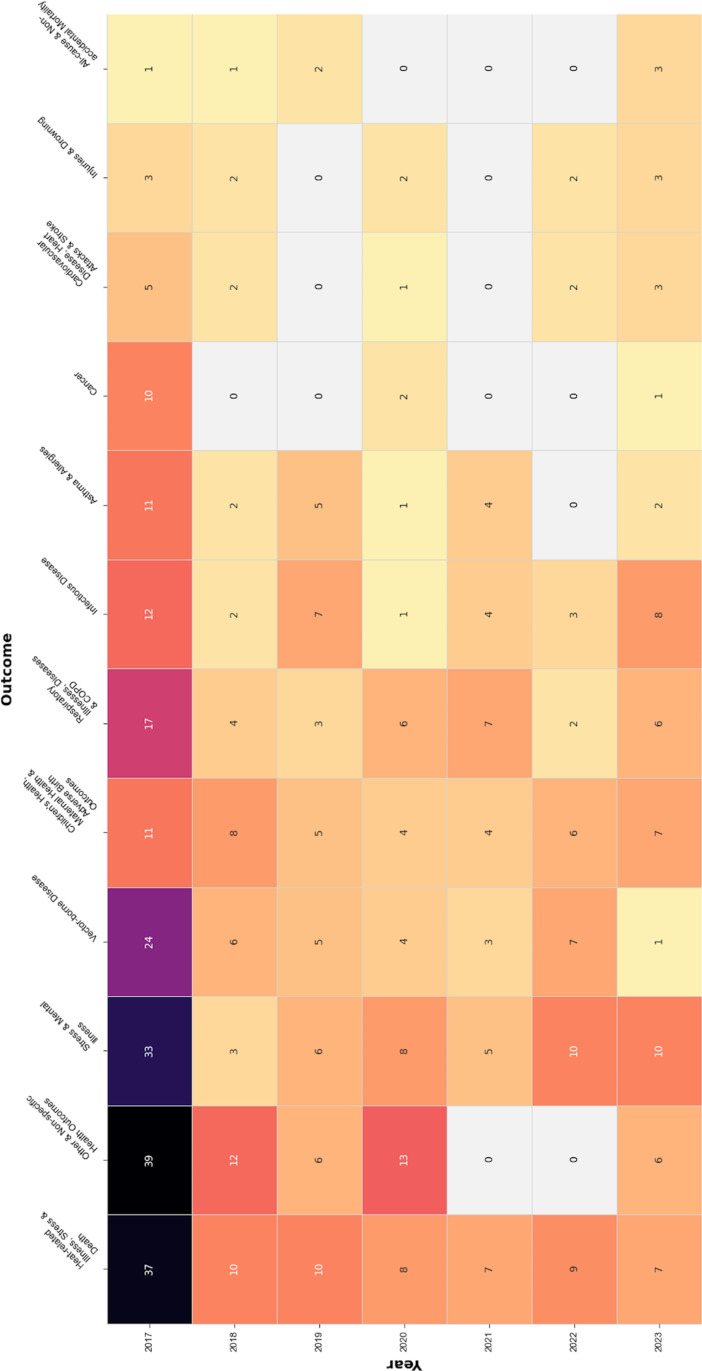
Heat map, discussed health outcomes in APHA 2017-2023 climate change-related abstracts (by year).

**Fig 5:**
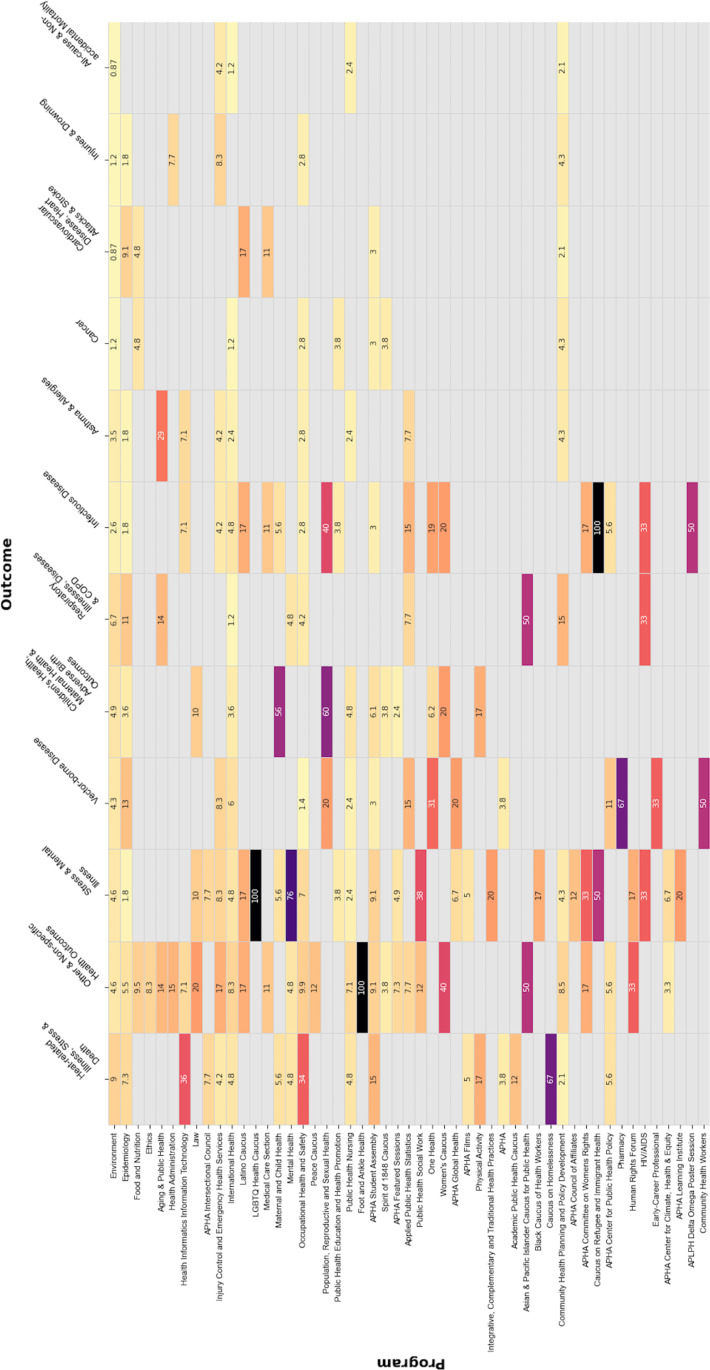
Heat map, discussed health outcomes in APHA 2017-2023 climate change-related abstracts (by program).

While most abstracts did not discuss specific health impacts of climate change, several abstracts focused on one or more health outcome categories (see Figs 4 and 5). The single most discussed category was *heat-related illness, stress & death*, followed by *stress & mental illness*, and *vector-borne disease*. *Heat-related illness, stress & death* was discussed in two-thirds of the climate-related abstracts included in the Caucus on Homelessness program and about one-third of those in the Health Informatics Information Technology and Occupational Health and Safety programs. The Environment program included abstracts discussing all health outcome categories, and *heat-related illness, stress & death* were present in the largest proportion (9%) of climate-related abstracts. *Vector-borne disease* was the outcome category with the largest proportion (13%) in the Epidemiology program, followed by *respiratory illnesses, diseases, & COPD* (11%) and *cardiovascular disease, heart attacks & stroke* (9%). Through time, *stress & mental illness* showed a growing presence, leading the number of abstracts in 2022 and 2023; notably, this theme was present in all climate-related abstracts identified in the LGBTQ Health Caucus program, in half of those in the Caucus on Refugee and Immigrant Health program, and in a third of those in the APHA Committee on Women’s Rights and HIV/AIDs programs.

## Discussion

The public health workforce faces many challenges, as we work to tackle climate change alongside many other major issues at a time when lack of funding and political support for such efforts persists. [16,17] As Fig 2 validates, the public health community is effective at naming and studying exposures and health outcomes related to climate change, but the discussion of the role of public health, workforce development, and policy and advocacy is limited, with slightly more attention dedicated to health communication and education. This is notable as individual behavior change is limited in addressing the impacts of climate change on health; whereas, policy, planning, and community action are critical. If this analysis of climate change discourse in a major public health annual meeting is reflective of the field’s discourse, more work is needed to prepare the public health workforce in developing, implementing, and assessing climate mitigation and adaptation strategies.

With the decreasing number and diversity of climate-related APHA abstracts in recent years, our analysis suggests that many opportunities persist for widespread health-centered climate action. However, “[r]esource limitations and lack of climate literacy among public health professionals are barriers to effective climate action,” as stated in a 2023 Environment Section abstract. Moreover, member communities whose programs included a minimal number of climate-related abstracts likely reflecting less engagement in discussing this public health issue, could challenge themselves to increase sharing on the policies or practices needed in their respective areas of public health to address climate change.

The need for an innovative, comprehensive, and coordinated way forward toward a strong and equitable response to climate change is possible [18] and, frankly, necessary, as many abstract authors suggested. For instance, among the abstracts from the most recent years examined in our analysis, we read statements such as:

*Interdisciplinary collaboration between nurses, engineers, and project managers created a space of mutual learning about clean energy projects and public health.*

- Public Health Nursing Section, 2023

*Collaboration between public health agencies and healthcare systems is crucial for climate preparedness and response.*

- Injury Control and Emergency Health Services, 2023

*Interdisciplinary collaboration within schools of public health is necessary to integrate climate change education into curricula.*

- Pharmacy, 2023

*Photovoice, a form of participatory action research, can help explore how children make sense of climate change, while inviting their critical reflection, arts-based expression, and action (a ‘bottom-up’ process).*

- Community Based Public Health Caucus, 2022

These abstract excerpts spell out many cross-sector, interdisciplinary, and collaborative public health practices that offer a way forward in the field’s ability to address climate change.

Within APHA, efforts are underway to engage expertise from across the field in addressing climate change. For instance, leaders in the Environment and Maternal and Child Health Sections have collaborated for years and have formed a cross-section working group to promote children’s environmental health in a variety of ways, including the development of core competencies and a Climate and Health Youth Education Toolkit. [19,20] Also, the Intersectional Council’s topical group on Climate Change and Health is working to engage all 33 APHA sections, emphasizing the importance of all domains of public health learning and working together for effective climate action. This member-led working group actively collaborates with APHA’s Center for Climate, Health and Equity staff to advance policy and action to tackle the climate crisis across public health disciplines. A major recent milestone spearheaded by the Center was the 2024 Climate, Health and Equity Summit, an annual two-day virtual event that aims to “bring together APHA members and partners from across disciplines … to explore the intersectionality of climate, health and equity; build community; and advance collaborative work in the pursuit of a healthier, more equitable future.” [12] Active engagement and collaboration between professionals, volunteers, and community members at large, whether directly engaged in public health or not, is critical to foster policy, planning, and community action to address the mounting challenges and health impacts posed by a changing climate.

Multi-solving approaches within and across sectors are underway to address climate-related health issues. [20–24] Multi-solving describes innovative approaches that address interconnected challenges or goals. In the context of climate change, this may look like creating renewable energy microgrids in a way that can support energy access, workforce development, and reliable healthcare services. For instance, between 2016 and 2018, the Center for Climate Change and Health of the Public Health Institute in Oakland, California, worked to enhance twelve urban local health departments’ (LHDs) capacity to address climate change, health, and equity through activities that fostered internal capacity building, vulnerability assessments, and community engagement, thereby integrating climate change into LHD practices and broader jurisdictional planning efforts, and shifting community dialogue toward including public health in climate change discussions. [21] More recently, the U.S. Centers for Disease Control and Prevention’s Climate-Ready States and Cities Initiative published “Climate and Health: A Guide for Cross-Sector Collaboration” with guidance for local and state public health agencies on how to engage with leaders in agriculture, emergency response and disaster preparedness, energy and utilities, healthcare, meteorology and climatology, sustainability and green design, transportation planning, urban planning, water utilities and management, and wildland management and forestry. [22] As authors of an abstract in the One Health Section’s 2023 program explained, “[t]he One Health concept can serve as an important educational and policy tool for public health professionals, enabling them to examine and address global health challenges, including climate change, during the 21st century.”

Our ability to make inferences about the climate change discourse at APHA was limited in several ways. Not every public health conference presentation needs to center on climate change but, given the urgency of the climate crisis and related health inequities, more attention is needed across the field. We acknowledge that the number of abstracts may not be the only metric of a robust discourse or climate response, as a few well-attended large sessions or keynotes could shift discourse as well, or better, than several smaller sessions. Our analysis focused on accepted abstracts; however, some sessions or presentations may have been canceled. Furthermore, our abstract collection did not include the finalized APHA program for 2023; hence, our analysis omits late-breaking abstracts in that year. While we reviewed all automatically identified abstracts for relevance to climate change, we relied on AI for theme detection. Further, some abstracts were short, which limited our ability to determine climate change relevance and themes. We did not attend all sessions, nor did we have access to the presentation materials for the talks of interest. Finally, while APHA is one of the largest public health forums, climate change discourse occurs in many other settings attended by public health scientists and practitioners.

Despite these limitations, this analysis provides useful information about the broad trends in climate change discourse within the public health community, and it offers a clear call for intra- and intersectional efforts while keeping environmental justice a priority. As other public health priorities inevitably and rightfully rise to the top, we cannot lose sight of the fact that climate change must remain a focus of all public health work and not just for those working in environmental health. Those working in academic, continuing education, community health work, or medical training must build climate and health-related literacy. For instance, those working to address disability or women’s rights, those supporting school-based health, and those serving as public health nurses and social workers— all have a role to play in protecting health in a changing climate. As Poet Laureate Ada Limón poignantly explains, “The world says, Once we were separate, and now we must move in unison.”

## Supporting information

S1 Text APHA Abstracts by Outcome & Topic_2025-0126 31925. (XLSX)
